# A mixed methods approach to adapting and evaluating the functional assessment of HIV infection (FAHI), Swahili version, for use with low literacy populations

**DOI:** 10.1371/journal.pone.0175021

**Published:** 2017-04-05

**Authors:** Moses K. Nyongesa, Antipa Sigilai, Amin S. Hassan, Janet Thoya, Rachael Odhiambo, Fons J. R. Van de Vijver, Charles R. J. C. Newton, Amina Abubakar

**Affiliations:** 1 Centre for Geographic Medicine (Coast), Kenya Medical Research Institute, Kilifi, Kenya; 2 Pwani University, Kilifi, Kenya; 3 Tilburg University, Tilburg, Netherlands; 4 North-West University, Potchefstroom, South Africa; 5 University of Queensland, Brisbane, Australia; 6 University of Oxford, Oxford, United Kingdom; University of California San Diego, UNITED STATES

## Abstract

**Background:**

Despite bearing the largest HIV-related burden, little is known of the Health-Related Quality of Life (HRQoL) among people living with HIV in sub-Saharan Africa. One of the factors contributing to this gap in knowledge is the lack of culturally adapted and validated measures of HRQoL that are relevant for this setting.

**Aims:**

We set out to adapt the Functional Assessment of HIV Infection (FAHI) Questionnaire, an HIV-specific measure of HRQoL, and evaluate its internal consistency and validity.

**Methods:**

The three phase mixed-methods study took place in a rural setting at the Kenyan Coast. Phase one involved a scoping review to describe the evidence base of the reliability and validity of FAHI as well as the geographical contexts in which it has been administered. Phase two involved in-depth interviews (n = 38) to explore the content validity, and initial piloting for face validation of the adapted FAHI. Phase three was quantitative (n = 103) and evaluated the internal consistency, convergent and construct validities of the adapted interviewer-administered questionnaire.

**Results:**

In the first phase of the study, we identified 16 studies that have used the FAHI. Most (82%) were conducted in North America. Only seven (44%) of the reviewed studies reported on the psychometric properties of the FAHI. In the second phase, most of the participants (37 out of 38) reported satisfaction with word clarity and content coverage whereas 34 (89%) reported satisfaction with relevance of the items, confirming the face validity of the adapted questionnaire during initial piloting. Our participants indicated that HIV impacted on their physical, functional, emotional, and social wellbeing. Their responses overlapped with items in four of the five subscales of the FAHI Questionnaire establishing its content validity. In the third phase, the internal consistency of the scale was found to be satisfactory with subscale Cronbach’s α ranging from 0.55 to 0.78. The construct and convergent validity of the tool were supported by acceptable factor loadings for most of the items on the respective sub-scales and confirmation of expected significant correlations of the FAHI subscale scores with scores of a measure of common mental disorders.

**Conclusion:**

The adapted interviewer-administered Swahili version of FAHI questionnaire showed initial strong evidence of good psychometric properties with satisfactory internal consistency and acceptable validity (content, face, and convergent validity). It gives impetus for further validation work, especially construct validity, in similar settings before it can be used for research and clinical purposes in the entire East African region.

## Introduction

Globally, the sub-Saharan African (SSA) region remains the most severely affected by the HIV/AIDS pandemic [1] and is home to 71% of people living with HIV/AIDS. With enhanced access to treatment and lowered mortality, attention is now shifting to the understanding of HIV-related co-morbidities and long-term effects, including the impact of illness and treatment on quality of life. In Africa, specifically SSA, few studies have focused on assessing the Health-Related Quality of Life (HRQoL) of individuals diagnosed with HIV infection [2] despite the fact that it is an important outcome in the course of HIV disease [3]. For instance, three studies examining the impact of Highly Active Antiretroviral Therapy (HAART) on HRQoL in South Africa found a significant association between HAART and improved physical, emotional and mental health [4–6]. However, these three studies employed only generic measures of HRQoL, some of which have been criticized for not capturing all domains relevant to HRQoL [7]. By the nature of their design, generic HRQoL can be applicable to anyone [8] with a more focus on the physical functioning and less attention to other domains related to the psychological and social experiences or adaptive capabilities of an individual [9]. Other than physical manifestations, existing evidence also shows that antiretroviral therapy, psychological and spiritual well-being, coping strategies and social support structures are important predictors of HRQOL among the HIV-affected population [10]. Therefore, such measures may end up being insensitive to the more specific aspects of HRQoL in HIV disease process. This lack of specificity may lead to either an underestimation or overestimation of HRQoL scores, thereby under-reporting or over-reporting the actual impact of HAART on HRQoL.

One of the major hindrances to investigating the HRQoL among people living with HIV (PLWHIV) is the lack of culturally appropriate and adequately standardized measures. To address this gap, we set out to adapt and evaluate the Functional Assessment of HIV Infection (FAHI) questionnaire for use in a rural setting in Kenya. We report a mixed-methods study comprising three stages: a scoping review, a qualitative study aimed at content validation, and a quantitative study investigating the psychometric properties of the adapted scale.

Quality of life is multi-dimensional and examines a patient’s own subjective evaluation of his or her well-being [11, 12]. Evaluating HRQoL of patients in HIV clinical trials and practice may provide important complementary information to disease parameters like CD4 cell count or viral load that evaluate HIV progression but do not capture the patient’s experience of treatment benefit [13]. To do this requires adequately validated and psychometrically sound measures of HRQoL in areas with a high HIV burden. For the majority of HIV-infected individuals, the attained longer life expectancy with antiretroviral use is more salient if they can sustain high (or at least acceptable) levels of HRQoL [14].

There are a number of both generic and disease specific psychometrically sound and adequately validated instruments designed to measure HRQoL in PLWHIV [15–21]. However, as there is no ‘gold standard’ for measurement of HRQoL in HIV disease [17], the usefulness and relevance of currently available instruments need to be evaluated [22]. We selected FAHI as our preferred measure among the HRQoL assessment instruments. The FAHI questionnaire is an HIV specific adaptation of the Functional Assessment of Cancer Therapy-General (FACT-G) questionnaire [13, 17]. It is a 47-item self-report questionnaire that assesses HIV-specific quality of life in five domains: Physical Well Being (PWB), Emotional Well Being (EWB), Functional and Global Well Being (FWB), Social Well Being (SWB), and Cognitive Functioning (CF). Our choice of the FAHI questionnaire was informed by several reasons: 1) it has been used in other cultural settings [23, 24]; 2) it is considered one of the most appropriate HIV-specific measures of the HRQoL of HIV-infected patients [25]; and, 3) it is a psychometrically sound instrument that captures multiple important dimensions of HIV/AIDS related quality of life in both clinical practice and clinical trials [22]. In a recent review, O’Brien et al [26] categorized the FAHI as a HRQoL instrument with accepted breadth (having at least one item/category represented in each of the four disability dimensions) and depth (having all possible categories/items represented in a given dimension). The FAHI is also relatively brief (takes about 5–15 minutes to administer) [27], is easy to score, is available in many languages and has been validated in many centers worldwide [22, 23]. Given these characteristics we set out to adapt the FAHI questionnaire to make it culturally appropriate and contextually relevant for use among Swahili speakers. In particular, we aimed to:

1. determine the perception of PLWHIV on the impact of the disease in their day-to-day life with the purpose of content validation of the FAHI;
2. evaluate the FAHI questionnaire for clarity of items and relevancy;
3. evaluate the internal consistency and validity (face, construct and convergent) of the adapted tool.

## Methods

### Study site

The study was carried out at the Comprehensive Care and Research Centre (CCRC) located within the Kilifi County Hospital at the Kenyan coast. The Centre provides antiretroviral medication and offers clinical care services including the management of opportunistic infections exclusively among the HIV-infected population. Other services offered include family planning, cervical cancer screening, nutritional counselling and care and also HIV testing and counselling for high risk population.

In Kilifi County, the overall adult HIV prevalence is at 4.4%, with prevalence that is higher among women (6.3%) than men (2.7%). Sexual transmission accounts for a majority of new HIV cases in this region with early sexual debut and low condom use as the key pointers [28]. HIV related stigma and lack of social support have been reported [29] as factors that may negatively impact quality of life in this setting.

A significant number of adult patients have limited if any formal education and are unable to adequately respond in written surveys or questionnaires, hence the need for interviewer administration of instruments.

### Ethical considerations

The Kenya Medical Research Institute National Scientific and Ethical Committees approved the study. Written informed consent was obtained from all study participants prior to participation.

### Study design

This study was part of a larger study investigating antecedents and correlates of poor outcome among vertically infected children and adolescents. A mixed-methods design was used consisting of three phases. In the first phase, a scoping literature review was carried out to document the psychometric properties of the FAHI and contexts in which it has been administered. The second phase was qualitative, aimed to address the content validity of the adapted instrument. Piloting of the adapted FAHI was also carried out in this phase and modifications to make it contextually relevant were carried out. In the third phase, the internal consistency, convergent and construct validity of the adapted FAHI were evaluated.

## Phase I: Scoping review

### Aim

To provide an in-depth understanding of the evidence based reliability and validity of FAHI as well as the geographical contexts in which it has been administered.

### Methods

We conducted a review using systematic methods to search the literature for relevant articles. The following terms were utilized as search criteria on various databases including PubMed, Google Scholar and PsycINFO: “FAHI AND Quality of Life,” “Quality of Life AND FAHI AND HIV,” “FAHI Questionnaire” and “FAHI.” In addition, we used the references cited in identified articles to retrieve other relevant published articles. We did not apply any date or language restriction in the scope of the search. As inclusion criteria, the published articles should have: i) reported reliability and/or validity of the FAHI; and ii) used the FAHI as a HRQoL measure in trials of efficacy of a drug or in other intervention studies.

### Results

**Fig 1:**
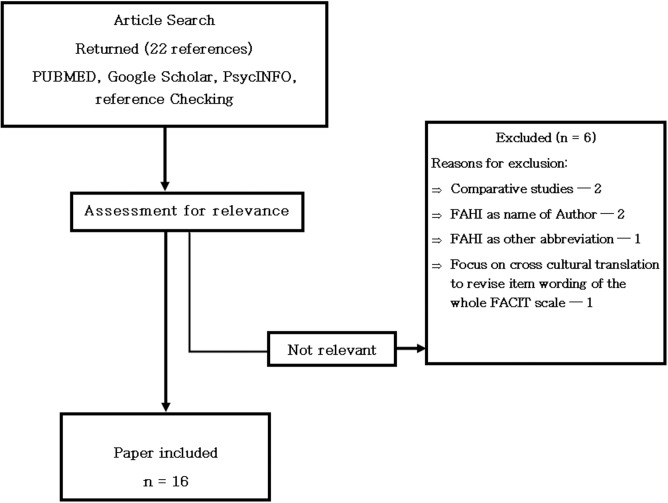
Flow chart of Scoping review.

We identified a total 22 articles, of which 16 met the inclusion criteria (see Fig 1 for the flow chart of the review and S1 Table for a list of the excluded articles). Table 1 presents the characteristics of the identified studies. A significant proportion of the published studies were conducted in the US (n = 13), while only 2 were from Africa. Seven out of the 16 reviewed studies reported psychometric properties. The internal consistency of the total scale in these studies was invariably excellent (Cronbach’s α range 0.91–0.92) [17, 22, 30–32]. The subscale α coefficients ranged from 0.72–0.92 with the exception of the cognitive function (CF) domain in two studies from South Africa whose α coefficients were slightly below the recommended 0.70 (Cronbach’s α values of 0.65 and 0.60) [13, 17, 22, 30–33]. None of these seven studies reported the test-retest reliability of the FAHI.

**Table 1:** Characteristics of identified studies.

						Key results/Findings (psychometric properties)							
Author (s)	Country	Year	Sample Size	Source		PWB α	EWB α	FGWB α	SWB α	CF α	FAHI	Established	Sensitivity
											Total α	validity	to change
Byrne S. & Petry N. [30]	USA	2012	n = 170	Single		0.85	0.89	0.82	0.70	0.70	0.92	Convergent	YES
												Discriminant	
Peterman A.H. et al [22]	USA	1997	n = 361	BIOQoL (n = 110)	$\}$							Divergent	YES
				VCU data (n = 39)		0.91	0.82	0.86	0.73	0.75	0.91	Construct	
				Multi-center trial (n = 212)									
Viala-Danten M. et al [13]	USA	2010	n = 1661	POWER trial (n = 565)		0.91	0.83	0.89	0.85	0.75	NR	Construct	NR
				DUET trial (n = 1,091*)		0.92	0.84	0.89	0.84	0.72	NR		
Cella D.F. et al [17]	USA	1996	n = 245	Stress sample (n = 110)	$\}$						0.91	Concurrent	YES
				BIOQoL English (n = 71)		NR	NR	NR	NR	NR	0.91		
				BIOQoL Spanish (n = 64)							0.92		
Feinberg J. et al [34]	USA, Canada	2011	n = 429	Single		NR	NR	NR	NR	NR	NR	NR	NR
	& Puerto Rico												
Earthman C.P. [33]	USA	2002	n = 25	Single		0.91 (7 Item)	NR	NR	NR	NR	NR	NR	NR
Hasanah C.I., Zaliha A.R.,	Malaysia	2010	n = 271	Single		NR	NR	NR	NR	NR	NR	NR	NR
Mahiran M. [23]													
Cianfrocca M. et al [35]	USA	2010	n = 73	Single		NR	NR	NR	NR	NR	NR	NR	NR
Rao D. et al [24]	Continental	2007	n = 273	Single		NR	NR	NR	NR	NR	NR	NR	NR
	United States												
	& Puerto rico												
Abrams D.I, Steinhart C.,	USA	2000	n = 221	Single		NR	NR	NR	NR	NR	NR	NR	NR
Frascino R. [36]													
Tomita A et al [31]	South Africa	2013	n = 160	Single		0.88	0.83	0.85	0.72	0.65	0.86	NR	NR
Tomita A et al [37]	South Africa	2014	n = 51	Single		0.92	0.80	0.83	0.73	0.60	0.90	NR	NR
Diamond C.,	USA	2010	n = 100	Single		NR	NR	NR	NR	NR	NR	NR	NR
Taylor T.H.,													
Anton-Culver H. [38]													
Cella D.F. et al [39]	USA	2010	n = 1203	DUET– 1 (n = 612)		NR	NR	NR	NR	NR	NR	NR	NR
				DUET– 2 (n = 591)									
Sikkema K.J. et al [40]	USA	2005	n = 235	Single		NR	NR	NR	NR	NR	NR	NR	NR
Winston A. [41]	Multi- centered	2010	n = 256	Single		NR	NR	NR	NR	NR	NR	NR	NR

BIOQoL-Bilingual Intercultural Oncology Quality of Life, VCU -Virginia Commonwealth University, FAHI–Functional Assessment of HIV infection, PWB–Physical Wellbeing, EWB–Emotional Wellbeing, FWB–Functional and Global Wellbeing, SWB–Social Wellbeing, CF–Cognitive Functioning, POWER–Performance Of TMC114/r When evaluated in treatment-Experienced patients with protease inhibitor Resistance. DUET- a randomized, double-blind, placebo-controlled, phase III trial examining the efficacy, tolerability, and safety of TMC125 in treatment-experienced patients. NR–Not Reported

* Number of patients with all sub scale scores available

A study by Peterman and colleagues [22] was the only one that reported the results of a factor analysis. Five items of the 44-item FAHI questionnaire had a factor loading less than 0.4, with 2 respiratory items “I have been coughing” and “I have been short of breath” having a poor conceptual fit with the factor on which they loaded most highly. The emotional wellbeing factor explained 11% of total variance, the functional wellbeing factor explained 15%, the social wellbeing factor explained 7%, whereas the cognitive functioning factor explained 10% of the total variance.

## Phase II: Adaptation and assessment of content and face validity

### Aims

Were to: (1) translate the FAHI questionnaire from English to Swahili; (2) elicit the perception of PLWHIV on how the disease has impacted on their day-to-day life for the purpose of evaluating the FAHI’s content validity; and (3) evaluate the questionnaire for relevancy, word clarity and content coverage. The evaluation sought to establish the content validity of the instrument, clarity of the items and relevancy of the items in capturing information related to the physical, emotional, functional, social and cognitive functioning domains of well-being. The questionnaire was forward translated by two independent translators after which a six-member panel was engaged in the harmonization and adjudication process. Structured open-ended questions aimed at capturing the perceptions of PLWHIV on the impact of HIV infection and its treatment on their life in the various domains of well-being were developed. Four questions evaluating the adapted FAHI questionnaire for relevancy, word clarity and content coverage were included at the end. In evaluating for content coverage of the adapted FAHI, participants were asked whether, in their opinion, they thought the questions asked covered the challenges they encounter in the domains of physical, functional, emotional, social and cognitive functioning.

### Sample

A sample of 38 study participants receiving services at the CCRC was randomly selected to participate in the qualitative study. Male and female participants were recruited who were aged over 18 years; provided consent for their participation; were aware of their HIV status; and on antiretroviral medication. The same participants were involved in piloting of the translated FAHI questionnaire. Table 2 shows the demographics of participants. Majority of the respondents were females (n = 27, 71%), married (n = 22, 58%); only 29% of the respondents had any form of secondary level education.

**Table 2:** Characteristics of study participants—phase II and III.

	Phase II N = 38	Phase III N = 103
**Mean Age [SD] (in years)**	40.89 (10.42)	37.37 (7.76)
**Gender**		
Female	27 (71.1%)	97 (94.2%)
Male	11 (28.9%)	6 (5.8%)
**Level of education**		
None	8 (21.1%)	33 (32.0%)
Primary	19 (50.0%)	54 (52.4%)
Secondary	11(28.9%)	12 (11.7%)
College	0 (0%)	4 (3.9%)
**Marital status**		
Divorced	1 (2.6%)	0 (0%)
Married	22 (57.9%)	59 (57.3%)
Separated	7 (18.4%)	23 (22.3%)
Single	4 (10.5%)	4 (3.9%)
Widowed	4 (10.5%)	17 (16.5%)

### Methods

An interview was conducted in a private, quiet room using structured open ended guidelines. The responses were recorded using either a voice recorder (with the participant’s permission) or through note-taking (when the participant refused to be recorded). Both the translated 5-point and 3-point Likert versions of the FAHI questionnaire were piloted after each interview session. The administration of the 3- or 5-point scale was carried out on different participants selected randomly from the recruited sample. If in one interview the 3-point version was piloted, the 5-point version would be administered in the subsequent interview.

### Procedure

The interviewer first presented a description aimed at explaining the purpose of the interview to participants. The interviewer then asked open-ended questions aimed at understanding the participant’s perception of the impact of HIV infection on the life of PLWHIV in general. The full version of the FAHI was then administered after which participants were requested to evaluate each item. Participants were also requested to make suggestions on any other information that might have not been captured by the items but considered to be of great importance among PLWHIV. They were also requested to single out items they thought were not relevant.

### Results

We summarized the participants’ responses to questions targeting evaluation for relevancy, word clarity and content coverage of the adapted FAHI. When evaluating item relevance, most respondents (34 out of the 38, 90%) said they were satisfied with the questions asked and that the questions were relevant:

“They are good and will give you what you want to know about challenges people living with HIV undergo”

(Married, female participant, 32 years old)

A few of the participants (4 out of the 38) expressed concerns with some items in the questionnaire. The items “I am enjoying the things I usually do for fun”, “I am content with the quality of my life right now”, “I feel sexually attractive” in the Functional Wellbeing (FWB) domain and “I am satisfied with my sex life” in the Social Wellbeing (SWB) were in their opinion not appropriate to ask patients already bearing the burden of living with HIV infection.

Participants were asked whether they understood the questions after they completed each sub-scale. This was done so as to evaluate word clarity of the questionnaire. Most respondents (37 out of 38) reported understanding the questions. Only one of the respondents said:

“…a few are difficult to understand”

(Separated, male respondent, 42 years old)

A number of participants had difficulties distinguishing the options of “A little bit,” “Somewhat” and “Quite a bit” on the 5-point Likert scale. All participants reported that the questions covered the issues faced in these domains. Most of them were able to remember some of the questions asked and quoted such in explaining their level of agreement. None of the participants had any suggestions of what had been left out of the questionnaire which would have been important to ask.

The results of the piloting informed our decision to use a 3-point, rather than the 5-point Likert scale during phase three as this would ease comprehension of the rating options. The choice of the 3-point Likert may make our data more valid given the difficulties of administering high point Likert scales to low-literate population reported elsewhere [42]. Given that only a small percentage of our participants expressed concerns with some of the questions a decision was made to retain all the items in the original questionnaire.

#### Perception of PLWHIV on how the disease has impacted on their day to day living

FAHI items try to establish the quality of life in five domains of well-being by scoring a response using a Likert scale. In establishing the perception of our study participants on how HIV infection has impacted their day-to-day living, we wanted to find out if they would report issues elicited by items in the questionnaire as a way of evaluating the content validity of the adapted FAHI.

In the process of data analysis, we grouped participants’ responses into four main themes: impact on physical, functional, emotional and social wellbeing. There were also other emerging issues brought up by participants as a result of living with HIV-infection which were not covered under FAHI items. We grouped these under a separate theme of emerging issues.

#### Impact on physical wellbeing

Under this theme, participants reported a number of issues as a result of living with HIV-infection. Poor state of health, lack of body energy, fatigue, loss of appetite, weight loss and comorbidity were the emerging sub-themes. A state of poor health was the most reported negative outcome with 18 (47%) of the 38 participants mentioning issues related to poor health.

“You get colds, at times you experience coldness, joint weakness…there is diarrhoea, cough. Your health becomes poor”

(Married, female participant, 35 years old)

With HIV-infection, the body becomes weak, with difficulty in engaging in strenuous activities. This can explain why five (5) out of the 38 participants (13%) reported that they were easily fatigued especially when engaged in work or in an activity:

“I see work as a challenge, there is no work I feel I can comfortably do because I get tired easily”

(Single, female participant, 39 years old)

The participants perceived that HIV-infection has led to poor appetite, lack of energy and weight loss. Of the 38 respondents, 5 reported lack of body energy, 5 mentioned poor appetite while 4 talked of weight loss, resulting from living with HIV infection. A lack of physical energy, poor appetite and weight loss seem intertwined and to be occurring concomitantly:

“When you lose weight, you also lack body energy, you might also end up not eating”

(Separated, female participant, 37 years old)

Despite the aforementioned impact of HIV-infection on physical wellbeing, some of the participants pointed out that antiretroviral medication has led to great improvements in overcoming or reducing the negative impact of HIV infection:

“Medication is very important. So long as one follows instructions they will see the change because the initial state of frequent colds will be no more”

(Married, male participant, 52 years old)

#### Impact on functional wellbeing

In the functional well-being, living with HIV infection seems to have a notable negative impact on mainly the work or Activities of Daily Living (ADLs) of the infected individuals. Twenty-seven (27) out of the 38 participants (71%) reported that living with the infection interfered with their normal performance at work or other ADLs in one way or another. Reduced work-rate was reported as an outcome of being infected with HIV:

“You cannot do a lot of farm related work because of pain in various body parts. You fail to be as productive as before and more often you cannot strain in work”

(Married, female participant, 38 years old)

“Living with HIV affects the lives of infected individuals because having the infection means one’s activities would not go as planned, for instance one cannot be as productive at work as they were initially…sometimes, one is so sick and unable to continue with work”

(Married, female participant, 27 years old)

“Work overwhelms me; I am unable to do it as I initially used to”

(Single, female participant, 34 years old)

All individuals, whether they were formally employed, did casual labor or engaged in ADLs at home or the farm, seemed to be affected. For those who were formally employed, they had to seek permission from their bosses so as to attend their clinic appointment or go to the clinic to pick medication which may involve disclosure, and if granted, the time to go for medical appointment was often limited. Others were forced to be absent from work so as to honor their scheduled medical appointments or quit their jobs in cases where the condition deteriorated.

“If one is employed, like I am, when I need to go for my scheduled appointment, it becomes a challenge. It forces one to disclose to the boss…sometimes one’s condition can deteriorate to a point they can no longer continue working”

(Married, female participant, 27 years old)

“You may not have time to go to work when the time to go for medication is due. You cannot fail to go collect the medication, you would rather absent yourself from work”

(Separated, male participant, 50 years old)

#### Impact on emotional wellbeing

Participants reported experiencing emotional distress as a result of living with HIV infection. ‘Thinking a lot’ (a metaphor closely associated with depression in the cultural context of the study), fear of spreading the infection, stress and stigma were the sub-themes that emerged. Of the 38 participants, 10 (26%) reported to be ‘thinking a lot’ ever since they received a diagnosis of HIV infection. The thoughts of living with a disease with no cure, taking lifelong medication and the uncertainty of the future state of health were some of the reasons for the reported emotional disturbance:

“…when one becomes infected with HIV, the problem is that one will think a lot most of the time because you are taking lifelong medication”

(Separated, male participant, 42 years old)

“Thoughts are inevitable because the outcomes of this disease condition in the future are unknown”

(Single, female participant, 34 years old)

Participants also reported feeling stressed.

“If compounded with poverty, HIV can be very stressing; there is fear of unknown upon diagnosis…stress of being known to be suffering from HIV”

(Married, male participant, 32 years old)

HIV related stigma was also described by some participants.

“Being afraid, stigma it is…’”

(Married, male participant, 66 years old)

#### Impact on social wellbeing

From the participants’ responses, six sub-themes clearly emerged in social interactions. Participants talked about isolation, discrimination, challenges of disclosure, poor social relationships, separation and divorce and problems in sexual relations.

Twenty (53%) of the 38 respondents reported fear of isolation by a family member, a friend or other members of the community because of living with HIV-infection. This is one of the factors that contributed to reluctance from infected individuals to disclose their status to most people according to some participants:

“It is usually disturbing because when one knows you are HIV-infected, he or she will isolate you, they will be uncomfortable with you…even your own family will isolate you. Neighbors too, it is only a few who will not, but majority will”

(Married, female participant, 32 years old)

A few of the participants (9 out of the 38) talked of discrimination. Some feared to be discriminated against and as a result they would rather not disclose their status based on past experiences. In their opinion, the participants explained that discrimination can be at home, at work or in business:

“They are discriminated, varies with the family. Like in my family, they will discriminate me that is why I have not disclosed to my mother or father…because my sister had this condition and was discriminated to an extent that she no longer goes home, she stays in Nairobi”

(Married, female participant, 32 years old)

“When you are selling things like buns or vegetables, others will discriminate your goods”

(Married, female participant, 38 years old)

Seventeen (45%) of the respondents reported difficulties of disclosing their HIV status to family members, friends, relatives or other people. Some of the highlighted reasons for non-disclosure included fear of stressing or depressing family members, fear of being isolated or discriminated upon, others might publicize one’s condition after they are told or there may be poor social relations afterwards:

“As for my neighbors, there may be only one who knows my condition, because you know there are others you will tell and then they tell everyone else, and that is not good”

(Widowed, female participant, 30 years old)

“It can cause problems, you fear telling them, like my mother [I fear letting her know] she might die of [high blood] pressure…”

(Single, female participant, 39 years old)

The relationship and interaction with family, friends, neighbors and others was poor or unstable as a result of living with HIV-infection as explained by 20 (53%) respondents. Some participants explained how certain names are used to describe them, how unfriendly others have become, the hatred shown to them, incidences of abusive comments and being despised. These poor social relations explain why PLWHIV become isolated or discriminated and why they sometimes find it hard to disclose their status:

“There are a lot of terms they use [to describe one who is HIV- infected], they say ‘you have been bitten by a goat’, ‘you are living a bonus life’…”

(Married, female participant, 47 years old)

“In my opinion, it depends on who will know about the condition. Others will hate you, others will not associate with you because they fear you might infect them. So it is better not to disclose in my opinion, maybe you only tell those you trust will not back bite you”

(Married, female participant 32 years old)

Living with HIV-infection has also destabilized families. It has resulted into separation or divorce. Blame games (of promiscuity) occur, there is uncertainty and distrust which destabilizes the family and leads to separation or divorce sooner or later:

“Affects relationships as it brings doubts and uncertainty; there can be blame games or even separation among spouses”

(Married, male participant, 32 years old)

There appears to be a problem with the way spouses or couples relate intimately as a result of living with HIV infection. Some of the participants who opened up about their sexual relations in the interview session mentioned challenges of sexual relations with their partners:

“In the family, there is a challenge between husband and wife because for them to be safe in an intimate relation, one of them is reluctant to use protection, you will get the wife is willing to use protection but the husband is unwilling”

(Married, female participant, 27 years old)

#### Emerging issues

Under this theme, participants described the challenges of getting food to meet the nutritional requirement of living with the HIV-infection, lack of job opportunities and the impact that HIV-infection has on societal development. Of the 38 participants, 3 (8%) felt that obtaining food is a problem and yet it is nutritionally recommended that they eat at least five times a day:

“The main impact for those living with HIV is food. This food, most are not in a position to get that which they are supposed to take. It is recommended that one eats at least five times a day but one can even fail to have a single meal and is forced to wait until evening”

(Separated, male participant, 50 years old)

“The general problem is that one takes medication and does not eat. I see that a problem”

(Widowed, female participant, 45 years old)

While HIV medication is acknowledged to enable participants be in a position to work, 2 of the 38 participants (5%) describe lack of jobs as a challenge. This has also contributed to the challenge of meeting the nutritional demands of living with HIV-infection:

“For sure, [HIV] medication gives us energy and we can even be able to work, but you find that there are no jobs”

(Widowed, female participant, 45 years old)

“There is a problem when it comes to jobs, sometimes when you don’t have a job, you lack food”

(Widowed, female participant, 52 years old)

Two out of the 38 participants described how the developmental progress of a society is affected when many people are infected or fall ill and as a result are unable to work:

“Everywhere, if in a society there are many ill people [HIV-infected], it shows that development will not be there because they will not be able to work and also the children being born might also be infected if necessary precautions are not taken. Therefore, such a society will derail in terms of development. Development is slowed down”

(Married, female participant, 27 years old)

In summary, items in four of the five subscales of the adapted FAHI questionnaire covered most of the issues perceived by the participants to be the impact of living with HIV infection. Participants did not report any issues under the three items of the cognitive functioning subscale of the questionnaire. The fact that issues raised by participants are covered by most of the items in the adapted FAHI questionnaire supports its content validity as a measure of HRQoL in this setting. Based on the experiences of the cognitive interviews, we modified the 5 point Likert scale into a 3 point Likert scale.

## Phase III: Psychometric evaluation

### Aim

To evaluate the psychometric properties (internal consistency, construct and convergent validity) of the adapted FAHI questionnaire.

### Sample

The FAHI Questionnaire was administered to a sample of 103 randomly selected study participants registered at the CCRC who met the following inclusion criteria: i) 18 years of age or above; ii) HIV-positive with a known diagnosis; iii) on antiretroviral medication; iv) parents to children in the age brackets of 3–5 or 12–17 years, regardless of whether the children are HIV-infected or not; v) voluntarily consented to participate in the study after being informed about the study. The mean age (SD) for the sample population, was 37.4 (7.78) years. Table 2 summarizes the demographic characteristics of the study participants. Majority of the participants were female (94.2%), and married (57.3%).

### Procedures

Eligible study participants were approached and the study objectives were described to them in detail. Those who consented their participation were recruited into the study.

### Measures administered

Data were collected by means of interviewer administered questionnaires.

#### FAHI Questionnaire

In the adapted measure individuals score on a three-point scale of 0 (not at all), 1 (somewhat) and 2 (very much). Forty-four items are scored, yielding total scores that range from 0 to 88, with higher scores indicating better QOL.

This adapted Swahili version of the FAHI is a preliminary one. Further validation work is being conducted with the approval of Functional Assessment of Chronic Illness Therapy (FACIT) who hold the copyright for the FAHI questionnaire (including any translated version). Anyone interested in the final Swahili version of the FAHI will be able to access it from FACIT (http://www.facit.org/) once the validation process is complete.

#### Shona Symptoms Questionnaire (SSQ)

The *Shona Symptoms Questionnaire (SSQ*) [43] was administered at the same time to establish the convergent validity of the FAHI questionnaire. The SSQ is a 14-item screening tool, based on a yes/no response, for common mental disorders including depression, generalized anxiety and somatic symptoms. It was developed and validated in Zimbabwe [43] using exemplary cross-cultural methods and it integrates local idioms and internationally recognized items for emotional distress.

#### Socio-demographic measures

Information on age, gender, level of education and marital status was obtained from the study participants. Age in years; sex (male or female); level of education (none, primary incomplete, primary, secondary incomplete, secondary or college) and marital status (Never married, married, separated or widowed).

### Results

To evaluate internal consistency, we used Cronbach’s alpha. Table 3 provides information on the means, variances, standard deviations and Cronbach’s α coefficients at the sub-scale level. All except one sub-scale had acceptable internal consistency levels. The CF sub-scale had a low coefficient of 0.55, way below the recommended 0.70 [44], which may be accounted for by the its small number of items (n = 3). Three items demonstrated a negative inter-item correlation in this initial analysis: ‘I have accepted my illness’ (in FWB, -0.04), ‘I worry about spreading my infection’ (in EWB, -0.07) and ‘My family has accepted my illness’ (in SWB, -0.08). Nonetheless, we did not exclude these three items as there was minimal improvement in the internal consistency after their deletion.

**Table 3:** Means, SD, Cronbach’s α and correlation for the adapted FAHI (n = 103).

Subscale	N of items	Mean	SD	Score Range	Cronbach's α	95% CI	Correlation with SSQ
Physical wellbeing (PWB)	10	14.77	3.82	0–20	0.78	0.71–0.84	*-*.620**
Functional & Global wellbeing (FWB)	13	21.09	3.69	0–26	0.71	0.62–0.79	*-*.336*
Emotional wellbeing (EWB)	10	15.05	3.32	0–20	0.66	0.55–0.75	*-*.462**
Social wellbeing (SWB)	8	10.28	3.42	0–16	0.67	0.57–0.76	*-*.332*
Cognitive functioning (CF)	3	4.73	1.38	0–6	0.55	0.37–0.68	*-*.451**

SSQ: Shona Symptoms Questionnaire

*. Correlation is significant at the 0.05 level (1-tailed).

**. Correlation is significant at the 0.01 level (1-tailed).

We used factor analysis, with principal axis factoring, to explore the dimensionality of the scale. Since we had a conceptual understanding of the possible number of factors, we conducted the analysis per subscale, each time obtaining a one factor solution. For all the subscales, the Kaiser-Meyer-Olkin measure of sampling adequacy value was above the recommended minimum value of 0.5 and therefore we proceeded with the factor analysis. The value for Bartlett’s test of sphericity was also highly significant for each subscale *(p <* .*001)* suggesting that there were correlations in the dataset appropriate for factor analysis. Table 4 shows the loading of items on each subscale factor of FAHI whereas Table 5 shows the loadings using subscale scores.

**Table 4:** Factor loadings of adapted FAHI subscale items.

Subscale and its items	Factor loadings
**Physical wellbeing** (KMO = 0.713)	
Feeling fatigued	0.782
Feeling weak all over	0.688
Getting tired easily	0.693
Lack of energy	0.551
Having pain	0.538
Feeling ill	0.453
Having nausea	0.381
Side effects of treatment	0.349
Trouble meeting the needs of my family due to physical condition	0.319
Spending time in bed	0.286
**Functional Global wellbeing** (KMO = 0.677)	
Work fulfillment	0.604
Enjoying life	0.590
Content with the quality of my life	0.556
Enjoying things done for fun	0.462
Motivation to do things	0.422
Satisfaction in coping with illness	0.421
Ability to work	0.416
Hope for the future	0.342
Appetite	0.339
Sexual attractiveness	0.325
Sleep	0.311
Loss of hope in fighting illness	0.245
Acceptance	0.184
**Emotional wellbeing** (KMO = 0.68)	
Worry about condition worsening	0.646
Worry about death	0.623
Nervousness	0.571
Embarrassment	0.499
Unhappy with appearance	0.501
Sadness	0.391
Concerned about the future	0.362
Worry about the effects of stress on illness	0.248
Disclosure	0.231
Worry about spreading the infection	0.147
**Social wellbeing** (KMO = 0.582)	
Emotional support from family	0.655
Support from friends	0.523
Satisfaction with sex life	0.490
Availability of help if needed	0.501
Closeness to partner or main supporter	0.430
Satisfaction with family communication	0.375
Closeness to friends	0.386
Family acceptance of the illness	0.248
**Cognitive functioning** (KMO = 0.624)	
Trouble remembering things	0.600
Trouble concentrating	0.543
Clear thinking	0.495

**Table 5:** Factor loadings of adapted FAHI using subscale scores.

Subscale	Factor loadings
Physical wellbeing	0.601
Functional Global wellbeing	0.772
Emotional wellbeing	0.771
Social wellbeing	0.516
Cognitive functioning	0.500

Our results indicated that each of these subscales could adequately be described by a one-factor solution using both the subscale scores and subscale items for the analysis. At the item level, all the items on a single subscale loaded on the respective subscale in the expected direction. Most items loaded well to the respective subscales. However, two items “acceptance of illness” in the FWB subscale and “worrying about spreading infection” in the EWB subscale had relatively poor factor loadings of 0.184 and 0.147 respectively, though in the expected direction. The factor loadings observed are indicative of an acceptable construct validity of the subscales of the FAHI questionnaire. The PWB factor explained 34.4% of the variance *(eigenvalue = 3*.*44)*, the FWB explained 23.6% of the variance *(eigenvalue = 3*.*06)*, EWB explained 27.7% of the variance *(eigenvalue = 2*.*77)*, the SWB explained 30.9% of the variance *(eigenvalue = 2*.*47)* whereas the CF explained 53.2% of the variance *(eigenvalue = 1*.*60)*.

Ideally an item level analysis to examine the factorial structure of the full FAHI scale should have been carried out. However, due to sample size limitation, we used the subscale scores to evaluate for the full factorial structure. Using PAF we observed that all the subscale scores strongly loaded on a single factor (>0.500) with 52.2% of the variance being explained. These results provide initial evidence of the construct validity of the FAHI.

A negative correlation between the SSQ score and the FAHI sub-scale would demonstrate convergent validity. A bivariate analysis using Pearson’s correlations confirmed these predictions with significant negative correlations coefficient with SSQ score seen in PWB, EWB and CF sub-scales (at the 0.01 significant level, 1-tailed) and in FWB and SWB sub-scales (at the 0.05 significant level, 1-tailed). Table 3 also shows the correlations between SSQ and the sub-scale scores.

To compare the association between sub-scale scores with age, level of education and marital status, various statistical tests were performed. Age and level of education had no statistically significant relationship with the sub-scale scores using Pearson’s correlation. The marital status variable options were regrouped into two groups; those with a partner (married) and those without a partner (single, separated and widowed) in identifying social support. Using independent *t* tests, there was a statistically significant difference in the Social well-being score; *t* (101) = -4.85, *p*<0.001 between those with a partner and those without, with computed effect size of -0.96.

## Discussion

We set out to culturally adapt and evaluate the psychometric properties of the FAHI Questionnaire, a health related quality of life measure in HIV/AIDS. We aimed to evaluate the internal consistency of the scale and its validity focusing mainly on content, face, convergent and construct validities.

In the scoping review, we observed that most of the studies (almost 82%) using the FAHI had been carried out in the USA. However, there were at least three studies from Africa and Asia, and these studies show that FAHI retained its good psychometric properties in their new context. Such information is important as the first step in the tool development process [45, 46] as it provides the initial evidence worthy spending time attempting to validate FAHI in different cultural contexts.

The internal consistency of the adapted FAHI questionnaire in our study was satisfactory. Although Cronbach’s alpha estimates higher than 0.7 are recommended for group comparison [13, 44], our alpha estimates in the SWB and EWB (0.66, 0.67 respectively) were slightly below the 0.70 estimate, yet remain within the acceptable range. Good alpha estimates were observed in the PWB, FWB and the overall scale. However, for the cognitive subscale the internal consistency was low (0.55). The low alpha may reflect a low inter-relatedness of items in this subscale or may partially be explained by the small number of items in this subscale. We favor the latter explanation since there is evidence that alphas are usually compromised in the presence of few items [47]. Additionally, our factor analysis indicates that the items had good factor loadings of 0.600, 0.543 and 0.495, an indication that they are interrelated. Moreover, our findings are consistent with earlier studies which generally reported internal consistency scores for FAHI above the acceptable cut-offs with the CF subscale usually having the lowest alpha coefficient [30–32].

In our factor analysis, most of the items loaded fairly well to their respective subscale (>0.30) indicating an acceptable construct validity of the adapted FAHI questionnaire. Only a few items loaded poorly, some of which like “I worry about spreading my infection” in the SWB subscale being found to have a poor fit with this scale in the original validation work [22].There was a significant correlation between our adapted FAHI questionnaire and the Shona symptoms questionnaire using the subscale scores, indicative of strong convergent validity. In the validation of FAHI among patients with drug and alcohol use disorders, Byrne and Petry [30] also reported similar results in which significant correlations between FAHI sub-scales and the psychiatric subscale of the addiction severity index measure (ASI) were established. Consistent with earlier findings [13, 30] we confirm the acceptable construct and convergent validity of the questionnaire.

In our study, we found that participants’ perceptions of the impact of HIV infection on their day-to-day living overlapped with most of the items in the FAHI questionnaire domains. Among the issues that participants mentioned were lack of energy, fatigue, loss of appetite, weight loss, work-related difficulties, fear of spreading the infection and problems of disclosure. Their responses appeared to be captured within the PWB, FWB, EWB and SWB domains of the FAHI questionnaire confirming the content validity of this questionnaire. The impact of the disease on cognitive functioning did not emerge during the qualitative interviews. There are three potential explanations for this. First, cognitive deficits were not experienced in this population. Second, the deficits were too subtle for the patients to recognize for themselves. Third, due to participant characteristics such as limited education backgrounds or being overwhelmed by various HIV related stressors, they were unable to relate problems in their higher level cognitive functioning to their disease condition. We do not favor the first explanation since cognitive decline has been shown to accompany HIV progression in Africa and other regions [31, 48, 49]. Similar to what has been reported in other fields, we prefer the other explanations. For instance, in a study on depression in developing countries, it was found that people may not spontaneously offer information on cognitive symptomatology even when they would respond to having these symptoms upon structured interviewing [50]. This demonstrates the need for structured measures that evaluate all dimensions of quality of life such as the FAHI. The face validity of the tool was confirmed by the reported satisfaction with the relevancy, content coverage and clarity of items during the piloting phase. Our findings are therefore consistent with other quality of life studies that demonstrate the good content coverage of the FAHI [17].

There was an association between marital status and the social wellbeing sub-scale score of the FAHI questionnaire. Some of the items in the social wellbeing subscale ask about social support and this correlation might be indicative of good social support. Olagunju et al [51], in their study on HIV/AIDs and psychological distress, found that being married seemed to be a protective factor against psychological distress. Being married is potential protective because one gets social, emotional and even financial support from their spouse. Based on their results, they recommended psychosocial intervention strategies in which single HIV-infected adults without adequate social support mechanisms are provided avenues for accessing the support they need to optimize their quality of life.

The strength of this study was the use of mixed methods to adapt and evaluate the adapted FAHI questionnaire. However, the study had several limitations. Most study participants were females, since this study recruited parents of preschoolers and adolescents participating in other ongoing studies. Those who happened to accompany the children were mostly mothers. The limited gender variability may limit generalizability. We therefore recommend that future studies utilize larger sample sizes for proper evaluation of internal consistency at the subscale level. The sample should also reflect gender balance where possible. As a cross-sectional study, we cannot confirm test retest reliability, an important facet of reliability if the measure is to be used to monitor treatment effects. We also cannot comment on the HRQoL trends of the study participants. We therefore recommend the use of longitudinal studies that would effectively describe the trend of HRQoL, whether there is an improvement, decline or stabilization over time especially in this era of ART.

## Conclusion

The adapted interviewer-administered Swahili version of FAHI questionnaire showed initial strong evidence of good psychometric properties with satisfactory internal consistency and acceptable validity (content, face, and convergent validity). It gives impetus for further validation work, especially construct validity, in similar settings before it can be used for research and clinical purposes in the entire East African region.

## Supporting information

S1 Table A list of the six excluded articles (DOCX)
