# Supplementary material for: A mixed methods approach to adapting and evaluating the functional assessment of HIV infection (FAHI), Swahili version, for use with low literacy populations
Source: PLoS One. 2017 Apr 5;12(4):e0175021. doi: 10.1371/journal.pone.0175021 (PMC5381886; doi:10.1371/journal.pone.0175021)
Supplement: S1 Table — (DOCX) [file pone.0175021.s001.docx]

| **Author (s)** | **Year** | **Title of Publication** | **Journal** | **Reason for exclusion** |
| --- | --- | --- | --- | --- |
| Davis EA & Pathak DS (1) | 2001 | Psychometric Evaluation of Four HIV Disease Specific Quality-of-Life Instruments | The Annals of Pharmacotherapy | A comparative evaluation of four HRQOL measures citing an article already included |
| Clayson DJ et al. (2) | 2006 | A comparative review of health-related quality-of-life measures for use in HIV/AIDS clinical trials. | Pharmacoeconomics | A review comparing various generic and disease specific HRQoL measures that have been used in HIV/AIDS studies |
| Fathimulla F. (3) | 2000 | Preliminary assessment of health impacts for the Newport Chemical Agent Disposal Facility. | Drug & Chemical Toxicology | Not relevant. FAHI abbreviated as Final Assessment of Health Impacts (FAHI). |
| Sqalli-Houssaini T. et al. (4) | 2005 | [Effects of anxiety and depression on haemodialysis adequacy]. | Nephrologie & therapeutique | Not relevant. One of the authors name is **Fahi** Z |
| Benamar L. et al. (5) | 2003 | [Nephroprotection in young type 1 diabetic patients treated with converting enzyme inhibitor]. | Presse Medicale | Not relevant. One of the authors name is **Fahi** Z |
| Lent L. et al (6) | 1999 | Using cross-cultural input to adapt the Functional Assessment of Chronic Illness Therapy (FACIT) scales | Acta Oncologica | Not relevant. Main focus was on cross cultural translation to rename item wording of the whole Functional Assessment of Chronic Illness Therapy (FACIT) Measurement System |

**S1_Table: A list of the six excluded articles**

**References**

1. Davis EA, Pathak DS. Psychometric evaluation of four HIV disease-specific quality-of-life instruments. The Annals of pharmacotherapy. 2001;35(5):546-52.

2. Clayson DJ, Wild DJ, Quarterman P, Duprat-Lomon I, Kubin M, Coons SJ. A comparative review of health-related quality-of-life measures for use in HIV/AIDS clinical trials. PharmacoEconomics. 2006;24(8):751-65.

3. Fathimulla F. Preliminary assessment of health impacts for the Newport Chemical Agent Disposal Facility. Drug and chemical toxicology. 2000;23(1):55-66.

4. Sqalli-Houssaini T, Ramouz I, Fahi Z, Tahiri A, Sekkat FZ, Ouzeddoun N, et al. [Effects of anxiety and depression on haemodialysis adequacy]. Nephrologie & therapeutique. 2005;1(1):31-7.

5. Benamar L, Laboudi A, Rhou H, Laouad I, Fahi Z, Bentata Y, et al. [Nephroprotection in young type 1 diabetic patients treated with converting enzyme inhibitor]. Presse medicale (Paris, France : 1983). 2003;32(14):638-43.

6. Lent L, Hahn E, Eremenco S, Webster K, Cella D. Using cross-cultural input to adapt the Functional Assessment of Chronic Illness Therapy (FACIT) scales. Acta oncologica (Stockholm, Sweden). 1999;38(6):695-702.
